# A Comprehensive Study of Hops (*Humulus lupulus* L.): Botany, Phytochemistry, Cultivation, and Industrial Uses—Brewing, Pharmaceuticals, and Cosmetics

**DOI:** 10.3390/plants15182817

**Published:** 2026-09-14

**Authors:** Miguel Á. Collado, Francisco José González-Minero

**Affiliations:** Department Plant Biology and Ecology (Botany), Faculty of Pharmacy, University of Seville, 41012 Sevilla, Spain; mcollado1@us.es

**Keywords:** beer, botany, cosmetic, hops, *Humulus lupulus* L., medical plants, useful plants

## Abstract

Research on the multifaceted aspects of hops remains an active area of research with numerous publications appearing in recent years. In this article, we present a comprehensive review integrating foundational literature with contemporary studies, primarily those indexed in the Web of Science and Scopus databases. We compile a broad range of information on the species, encompassing ethnobotanical and taxonomic data, including its taxonomic position, morphological characteristics, nomenclature, palynology, and karyology. This review also provides a synthesis of recent genome sequencing studies, research on plant-derived powders, and extensive phytochemical investigations, together with information on cultivation practices and diseases. Furthermore, it examines the industrial and economic applications of hops, highlighting their importance in brewing, their medicinal uses, and their incorporation into cosmetic formulations. Considerations regarding toxicity and the safety of hop extracts are also addressed. From both economic and human health perspectives, *H. lupulus* is a plant of remarkable versatility and significance. Therefore, sustained interdisciplinary research is essential to fully elucidate and harness its diverse potential. Ultimately, the primary objective of this work is to provide a comprehensive understanding of this species for researchers across the various disciplines and professional sectors mentioned above.

## 1. Introduction

With the advancement of and access to multiple scientific research tools, new characteristics and properties of numerous plants have been studied in recent decades to determine their use and suitability in food and beverages, pharmaceuticals, cosmetics, and phytoremediation, among other applications. Here we present an overview of (*Humulus lupulus* L.) to complement and integrate recent data from different perspectives. *H. lupulus* (hops), alongside *Cannabis sativa* L. (hemp), both members of the Cannabaceae family, were described together by Carl Linnaeus [1] in *Species Plantarum* (1753) (2: pages 1027–1028; 1973) as taxa within the *Dioecia Classis* and *Pentandria Ordo*. Both species were established as monotypic genus by Linnaeus himself. Hops should not be considered a food or a condiment in the traditional sense, as it is not edible by itself. However, it indirectly affects millions of people through beer, which is a widely consumed beverage, ranking just behind water and certain infusions such as coffee (*Coffea arabica*) or tea (*Camellia sinensis*) [2,3]. Even in regions where wine was historically more common, such as Mediterranean areas, beer consumption surpassed wine consumption during the 20th century, becoming the most prevalent alcoholic beverage [4]. Conversely, alcohol consumption habits are changing across generations; for instance, Gen Z tends to consume less alcohol than previous generations [5], probably because the drug intake habits are changing to benzodiazepines [6] or opioids [7].

Beer is, in fact, a very ancient beverage, mentioned in the Hammurabi Codex (around 1795–1750 BC), which established regulations for its price and trade: *If a tavern keeper does not accept grain according to its gross weight as payment for the drink, but accepts money, and the price of the drink is less than that of the grain, she shall be condemned and thrown into the water* [8,9]. We should be aware that the substance referred to as “beer” in antiquity evolved significantly over time; it likely consisted of fermented grains flavored with herbs, dates, or myrrh. Initially, hops were not used as an aromatizer in Egyptian or early Mediterranean beer [10].

There is limited archaeological evidence related to beer. The oldest evidence of hops used in beer comes from Pombia (Italy), around 550 BC, where *H. lupulus* pollen was found in a vase containing beer leftovers [11]. Beer began to be brewed in monasteries in Central Europe during the Middle Ages, and it was then, around the 8th century, that monks introduced hops as an essential ingredient to flavor the product, add bitterness, and inhibit microbial activity (regardless of whether this was intentional). Initially, these were wild hops that grew on the walls and fences of the monasteries. Its success was remarkable, and planned cultivation began [12]. In 1516, in Bavaria (Holy Roman Empire), Duke William IV enacted the Beer Purity Law (*Reinheitsgebot*), which stipulated that beer should contain only malted barley, hops, and local water, in order to regulate prices and protect consumers from cheap or harmful additives [12]. Yeast had not yet been discovered, so it was not covered by the law, although it was used unknowingly during barley fermentation. The practice of adding hops to the brewing process then spread throughout Europe and, subsequently, to its colonies, especially England [13]. In 1926, the Hop Research Center Hüll (*Hopfenforschungszentrum Hüll*) was established in Bavaria, Germany, as a research institute dedicated to studying advances in hop chemistry, breeding, and cultivation. This institution focuses on developing new and innovative hop varieties (but logically, without the use of genetic engineering) to meet the specific demands of the brewing sector [14].

From medical point of view, neither Dioscorides nor Galen mentions hops however, Pliny the mentions *Lupus salictarius* [sic.] in his *Historia Naturalis* as a plant that can be used for food purposes [15]. Some old beliefs about the medical properties of hops are now known to be true. We need to skip to the 8th century, when the Persian physician Mesuë the Elder (Yahya ibn Masawaih) describes hops as a vine with rough leaves similar to cucumbers with inflorescences with anti-inflammatory properties. In the 11th century, Hildegard of Bingen (1098–1179) described hops as a good remedy against black bile and melancholy [15]. She noted that decocting half an ounce provides diuretic, hypnotic, and sedative properties; while small doses are bitter but tonify the stomach [15]. The sedative effects of hops were later exploited, as pillows were sometimes filled with hop cones to fight insomnia [16]. In fact, hop pillows are still being used and commercialized today; they served as an alternative to opium derivatives as a sedative due to the absence of side effects like headaches, stomachaches, or constipation [16].

Given that there are already several reviews specialized in different aspects of *H. lupulus*, the main goal of this work is to achieve a balanced and comprehensive overview of its various aspects. The second goal is to present the most recent results, combining them with current bibliography. The developed aspects are:

Botanical knowledge of *H. lupulus,* taxonomy, botanical description, karyology, palynology, molecular genomics and phytochemistry.

Agricultural studies and diseases that threaten the plant.

Compilation of the importance of this species and its botanical and commercial varieties in beer brewing, as a medical plant and cosmetics.

For this review, several searches were conducted in eb of Science, Scopus and Google Scholar, returning hundreds of results. Articles were filtered to include only the most recent ones (2022–2026), obtaining 148 results under the “*Humulus lupulus* review” query. This was once again restricted to different knowledge areas: Pharmacology, Pharmacy, Vegetal Sciences, Science & Food Technology, Agriculture & Biochemistry and Molecular Biology ith 134 related documents. Finally, taking into account the proposed objectives, 75 relevant articles were selected; after reviewing them, those containing redundant information were discarded. Additionally, foundational classic literature and publications from official and governmental organizations were included, reflecting the plant’s significant economic and commercial importance.

## 2. Results and Discussion

### 2.1. Taxonomical Location and Botanical Description

#### 2.1.1. Cannabaceae Family

*Humulus lupulus* L. is a species belonging to the family Cannabaceae Martinov (1820). In earlier classifications, this family was in the Urticales order (subclass Hamamelidae) according to Cronquist (1988) [17], or in the subclass Dilleniidae following Takhtajan (2009) [18]. In the most recent phylogenetic classifications, the Cannabaceae are located within the Rosales order (Eurosids I, Fabids), specifically in a monophyletic subclade named Urticalean Rosids (formerly Urticales), which also contains Ulmaceae, Moraceae and Urticaceae families [19,20].

The Cannabaceae family is composed of herbs, shrubs, or deciduous trees, which are sometimes climbing and lack latex. Stems are erect or twining and may be either scabrid or armed with rigid climbing hairs, often featuring glandular trichomes. Leaves are palmately lobed or palmately compound, petiolate, serrate, and generally decussate, with triangular, persistent stipules. Flowers are arranged in complex, basically cymose inflorescences and are unisexual (monoecious or, more often, dioecious). The fruits are characterized as achenes or drupes [21]. The family is distributed throughout temperate and subtropical regions and has reported basic chromosome numbers of x = 7, 9, and 10. Cannabaceae comprise 170 species distributed across 10 genera (*Aphananthe*, *Cannabis*, *Celtis*, *Chaetachme*, *Gironniera*, *Humulus*, *Lozanella*, *Parasponia*, *Pteroceltis* and *Trema*) [21].

#### 2.1.2. *Humulus* L. Genus

The genus comprises annual or perennial herbs characterized by rightward-twining bines. They are armed with hooked trichomes (hairs) that allow the plant to adhere to and twist around other plants or available structures. Leaves are typically 3- to 5-lobed, but can have up to 7 lobes The adaxial surface is scabrid (rough due to cystolithic hairs), while the have abaxial surface is irregularly covered with hairs and resinous glands. Male inflorescences are paniculate, and female ones are cone-like or in the form of strobili, featuring numerous glands that secrete aromatic substances [22].

To review the taxonomy of this genus, the WFO Plant List [23] was consulted, as it provides a comprehensive and official list that attempts to unify botanical nomenclature, particularly because many synonyms or illegitimate names are frequently used as accepted specific names. This is a website whose lists are regularly reviewed and updated by experts. Within the genus *Humulus*, there is an ongoing debate regarding the exact number of species. Traditionally, the genus was composed of three well-known species [23]: (1) *H. lupulus*, (2) *H. scandens* (Lour.) Merr (sometimes known by its synonym *H. japonicus*) and (3) *H. yunnanensis* Hu. These two last species have been considered to be used in beer brewing, but only *H. lupulus* and its varieties are truly important for industrial or medical use [24]. To the present day, there are 37 names of plants in which *Humulus* appears, but only 7 of them are well knonow, these include, in addition to thosepreviously mentioned: (4) *H. americanus* Nuez., (5) *H. cordiifoloius* Miq [25], (6) *H. nexomexicanus* Rydb. and *H. pubescens* Tembrock (7) [26,27,28].

The genus *Humulus* spp. consists of herbs, perennial or annual, twining and dioecious [28]. The stems are coarse and 6-ridged or winged. Leaves are opposite, with well-developed leaves being ± cordate and 3- to 7(-9)-lobed [29]; terminal leaves are sometimes ± ovate and simple, featuring yellowish-brown resinous glands and dots on the abaxial surface [22]. Male inflorescences are laxly paniculated. In male flowers, the filaments are straight in the bud; female inflorescences consist of a conelike spicate cyme with imbricate, ddpersistent bracts that enlarge in the fruit and have entire margins [28]. Female flowers feature a thinly membranous calyx appressed to the ovary with entire margins; the ovary is is urrounded by the appressed calyx, and the styles are 2-branched with caducous branches. The fruit is a broadly ovoid achene with a persistent calyx appressed to it; the pericarp is crustaceous, and the embryo is spirally involute with narrow cotyledons [30].

#### 2.1.3. *Humulus lupulus* L. Species

**Figure 1:**
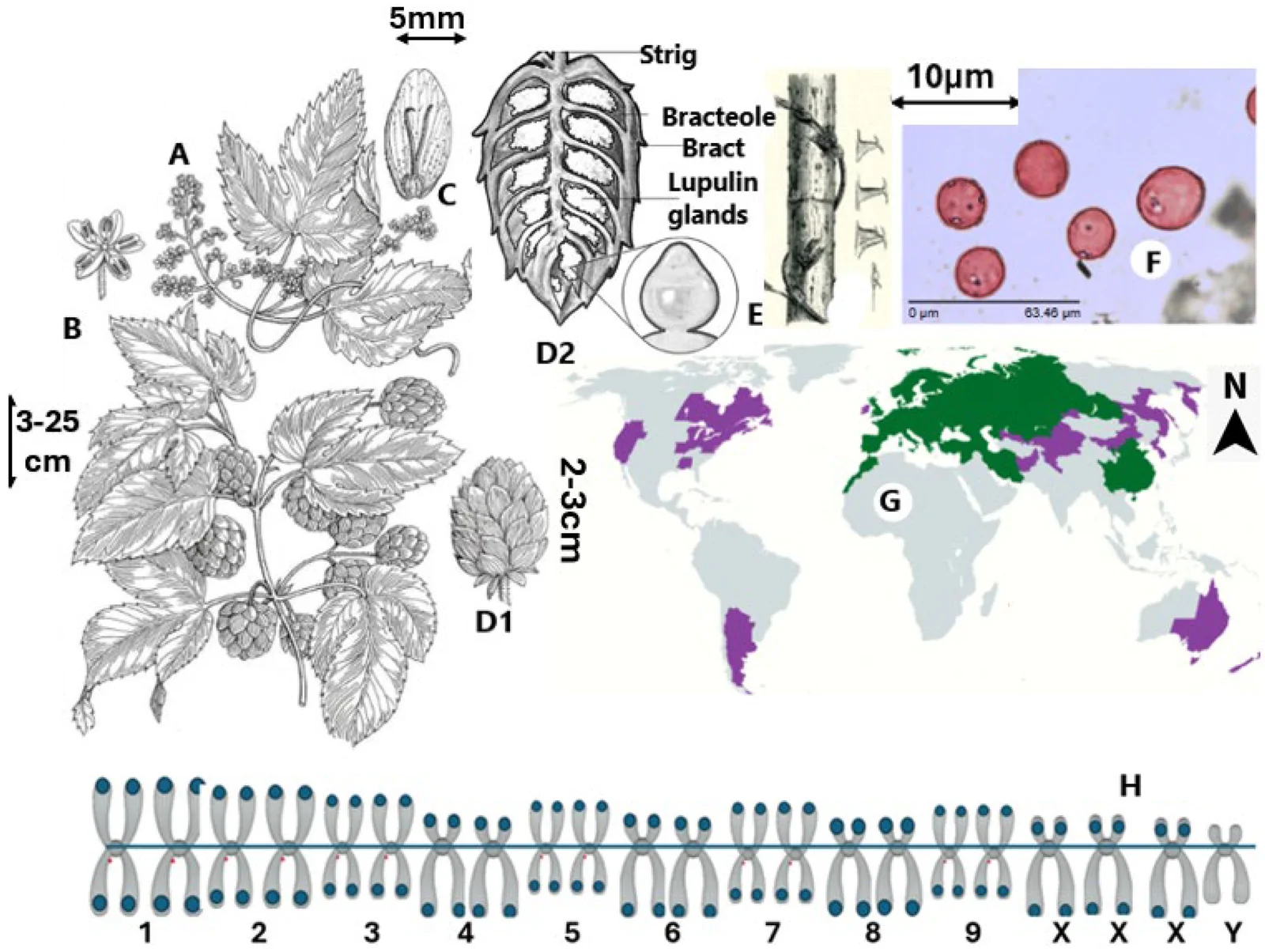
Botanical considerations regarding *H. lupulus*: morphology, palynology, distribution range, and karyology. (**A**) Female inflorescence; (**B**) Male flower; (**C**) Female flower; (**D1**) Female cone or strobili; (**D2**) Details of a female cone; (**E**) *Dextrosum* growth stem and bifid trichomes; (**F**) Pollen; (**G**) Distribution map (green native, purple introduced and cultivated); (**H**) Karyogram. Authors’ compilation, with images modified for scientific use or reproduced with permission from the original authors: [22,37,38,39,40].

Hops are perennial, rhizomatous, and dioecious plants. The annual aerial stems (bines) reach up to 5 (10) m in height and are dextrorse-twining (clockwise), armed with simple and two-forked trichomes used for adherence. The leaves are large and opposite, up to 18 cm long, with bifid stipules and petioles, featuring a broadly cordate base; they are palmately 3- to 7-lobed (oval-lanceolate) or occasionally unlobed, measuring 3 to 15 cm in length with dentate-serrate margins and mucronate teeth. The abaxial surface has glabrous or softly pubescent veins, while the adaxial surface is scabrid due to abundant cystolithic hairs. Male inflorescences are arranged in axillary bracteate panicles. Female inflorescences, commonly referred to as cones, are axillary, solitary, bracteate, and pedunculate, forming compact clusters (strobili) typically (1) 2–3 (6) cm in size. Male flowers consist of five sepaloid tepals and five stamens. Female flowers are imbricate and arranged in pairs around a central axis (rachis or strig) with overlapping green, foliaceous bracts and translucent bracteoles. The bracteoles directly envelop the tiny colorless flowers (an ovary with two filiform stigmas) and are accrescent, featuring numerous peltate glandular trichomes at the base known as lupulin glands, which develop as the inflorescence matures. The fruit is a 3 mm achene containing seeds with a spirally involute embryo and a small, oily endosperm. The chromosome count is 2n = 20, including two sex-determining chromosomes. The pollen is small (10–15 µm), spheroidal, and triporate, with a smooth surface (psilate) (Figure 1). The plant flowers in summer and fruits in early autumn in the Northern Hemisphere [22,29,31].

Common names: hop (English), hüfen (German), saut (French), lúpulo (Spanish), Saltur (portuguese), khmel (Russian).

The multiple botanical synonyms of *H. lupulus* are: *Waldensia lupulina* Lavy, *Lúpulo humulus* Molino., *Lupulus communis* Gaertn., *Lupulus amarus* Gilib., *Lupulus scandens* Justicia., *Humulus volubilis* Salisb., *Humulus vulgaris* Gilib., *Humulus lupulus* var. *lupulus*, *Humulus lupulus* var. *fengxianensis* JQFu, *Humulus lupulus* var. *sylvestris* Alef., *Humulus lupulus* var. *acrocarpo* Alef., *Humulus lupulus* var. *effusus* Alef., *Humulus lupulus* var. *spaltensis* Alef. [23]. In addition, there are around three hundred commercial varieties, classified into bitter, aromatic or mixed varieties [32].

Etymology: The term “hops” is derived from the Anglo-Saxon word *hoppan*, which means to climblikely referring to the plant’s nature as a bine. Regarding the genus name *Humulus*, some experts assume it stems from the Latin word *humus*, alluding to the rich, moist soil in which the plant thrives [31]. The specific epithet is derived from the Latin *lupus* (wolf), because the plant climbs and strangles osiers (*Salix* spp.) “as the wolf does to a sheep, a comparison attributed to Pliny the Elder [33]. On the other hand, *Humulus* is also considered a medieval Latin word that may have originated from Scandinavian (*hume*, *humal*) or Slavic (*schemli*, *chmely*) roots [27].

Habitat and distribution: There is a general consensus that the genus originated in East Asia, specifically China, because the three oldest species (*H. lupulus*, *H. yunnaensis* and *H. scandens*) are present in that region [34,35]. Wild plants are typically found in groves, floodplain thickets, and alder forests. This species has been introduced and cultivated below the equator between 35° and 55° S [36].

Observations: Naraine and & Small [41] studied the abaxial density of foliar glands. It was observed that North American hop varieties possessed an indumentum and a higher concentration of glands than their European and Japanese counterparts, with a gland density 2 to 4 times higher. This trait is recommendable as it increases resistance to herbivory.

The hop is an anemophilous plant; in certain regions, such as Turkey, it has been reported to account for up to 1% of the total atmospheric pollen [42]. This is a relatively low quantity because male individuals are typically avoided in commercial cultivation. This is not the case for its sister genus, *Cannabis*, whose pollen is usually prominent in aerobiological samplings when growing in the wild [43]. However, it remains difficult to distinguish between the pollen of these two genera unless their pores are measured (which are smaller than 1 µm in hops) [43]. Additionally, *H. lupulus* can cause dermatitis in some individuals who handle the plants; it is estimated that approximately 1 in 30 people are affected [44]. On the other hand, hops and *Cannabis* are botanical siblings that diverged from a common ancestor millions of years ago. Despite sharing a similar genetic architecture, including trichome development and pollination strategy, hops do not produce THC or CBD because the specific enzymes used for their synthesis are absent. For further information on this matter, please consult Aditi et al. [45].

Unlike most animals, plants do not have a pair of sexual chromosomes that determines sex. However, some dioecious plants (around 6% of all spermatophytes) [46] possess chromosome pairs that can be treated as allosomes. When Karlov et al. [40] unveiled the *H. lupulus* karyotype using *DAPI* markers (which bind strongly to adenine and thymine), they found that the karyotype is 2n = 20. These markers revealed signals near the telomere on one pair of chromosomes in a female plant, while in a male specimen, a short chromosome without a signal (the *Y* chromosome) was found (Figure 1).

At the molecular level, recent years have seen numerous studies utilizing molecular markers across various hop varieties to obtain genomic sequences and correlate them with gene expression. The goal is to identify and select for desirable secondary metabolites or [47,48,49]. This is not an easy task because the *H. lupulus* genome is large and complex, with a repeat sequence content of approximately 71.5% [50,51]. Padgitt-Cobb et al. [52] published draft assemblies of this genome (specifically for the *Cascade* variety), recovering more than 91% of essential (nSTBUSCO) genes. This genome has an estimated size of 2.8 Gb, which is more than three times the genome size of *Cannabis sativa* (0.808 Gb, 2n = 20). It is estimated that the genome contains over 80,000 gene models, with approximately 30,000 of them representing a high-confidence core set responsible for basic plant functioning [52].

### 2.2. Medicinal Applications and Phytochemistry of H. lupulus

The European Pharmacopoeia [53] includes 43 monographs on herbal drugs, establishing official quality criteria for various plant parts used for medicinal purposes. Among the specific texts for hops, the strobili are covered under the Latin name *Lupuli flos*. This monograph defines strict requirements for the dried female inflorescences of *H. lupulus*, setting thresholds to determine foreign matter, moisture content, and bitter active compounds to ensure safety and therapeutic efficacy.

**Figure 2:**
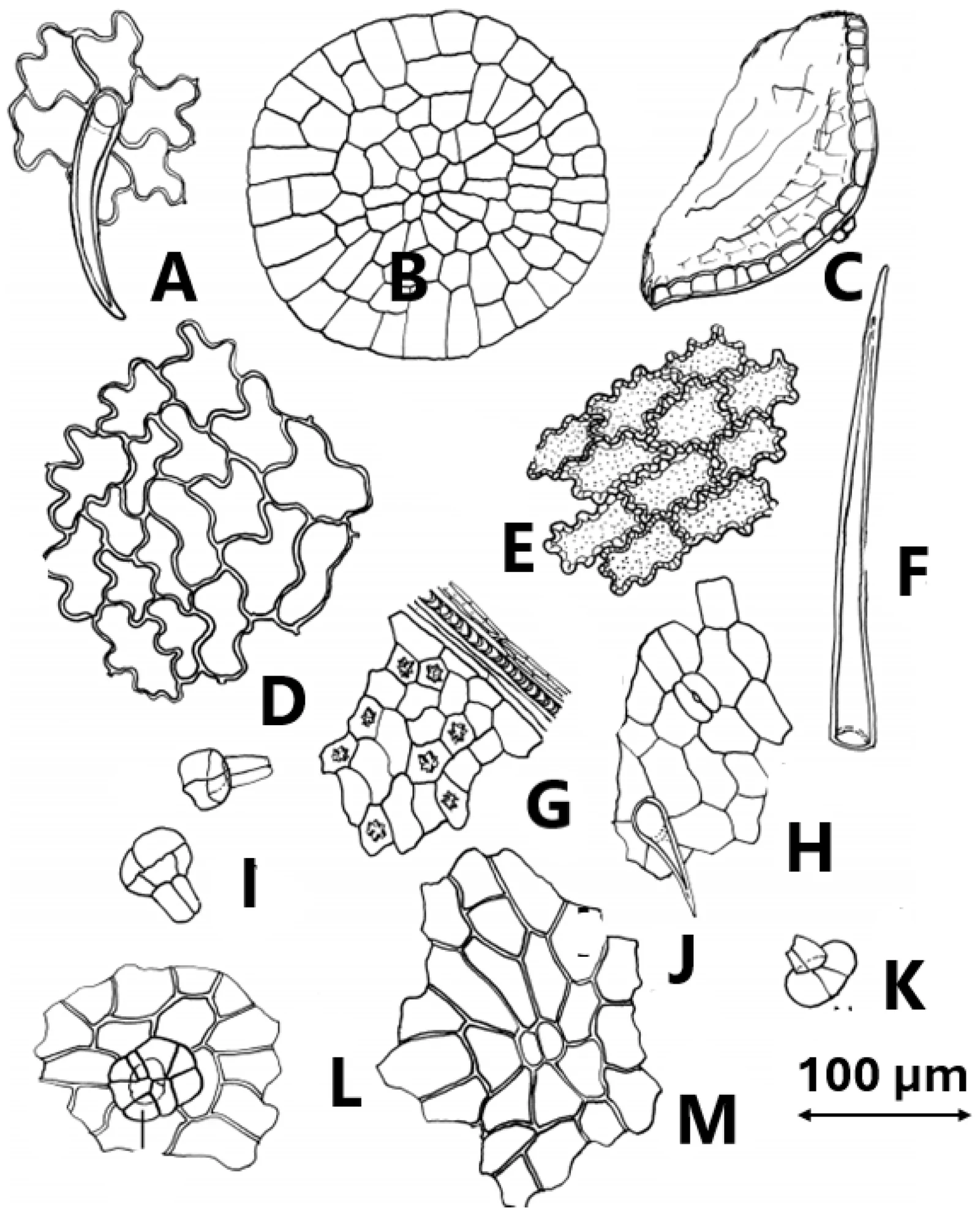
Analysis of the pulverized cones. Unicellular, conical, straight or curved non-glandular trichomes with thin, smooth walls (**A**) occurring on the epidermis (**E**,**G**); abundant, characteristic yellow-orange glandular trichomes with a short, bicellular, biseriate stalk bearing an expanded, cup-shaped base, 150–250 µm in diameter, consisting of a hemispherical layer of secretory cells. The cuticle is detached and distended by the accumulation of oleoresinous secretions. These trichomes are observed in frontal view (**B**) and lateral view (**C**); glandular trichomes, generally detached (**H**); glandular trichomes, generally detached, with a bicellular, biseriate stalk and a head composed of eight small cells (**I**); fragments of bracts and bracteoles covered with irregular, polygonal epidermal cells with sinuous anticlinal walls (**D**,**L**,**M**); fragments of elongated sclerenchymatous testa cells with thickened, striated walls and numerous pits (**F**); mesophyll fragments containing small calcium oxalate druses (**J**); and sparse anomocytic stomata (**K**). Royal Spanish Pharmacopeia (Ph. Eu.).

The method prescribed in the European Pharmacopoeia is based on the macroscopic appearance and morphological traits, as well as the characteristic odor of the strobili—generally whole and dried—which are then pulverized into a powder. To determine moisture and ash content, various tests are employed, including thin-layer chromatography (TLC). In the yellowish-green powder (the officinal powdered drug) dissolved in chloral hydrate, diagnostic microscopic elements can be observed using an optical microscope (Figure 2). Once the cones are pulverized, the resulting powder is analyzed [53].

Scientific interest in *H. lupulus* chemistry began approximately a century ago, driven by the industry and brewing organizations such as the European Brewery Convention (EBC) and the American Society of Brewing Chemists (ASBC). The table below shows the average quantitative analysis of chemical compounds from whole dried hop cones (comprising the rachis or strig, bracts, bracteoles, and lupulin glands). These constituents include primary metabolites such as proteins, monosaccharides, and cellulose, as well as secondary metabolites like resins, essential oils, and polyphenols [54]. This study of the proximate chemical composition (bromatological analysis) is frequently cited in the literature and has been compared with the results of subsequent, similar experiments (Table 1), which have revealed few significant differences from the initial findings, except with regard to resins and polyphenols. No additional studies were found for waxes and steroids. This variability may be attributed to the use of different analytical instruments or to the study of different hop cultivars [54].

Hop resins are classified into soft (yellow) and hard (dark and insoluble) resins; they are non-polar and can be divided according to their solubility in hexane. Furthermore, these resins can be dissolved in oils, alcohols, and non-polar solvents such as liquid carbon dioxide [57].

**Figure 3:**
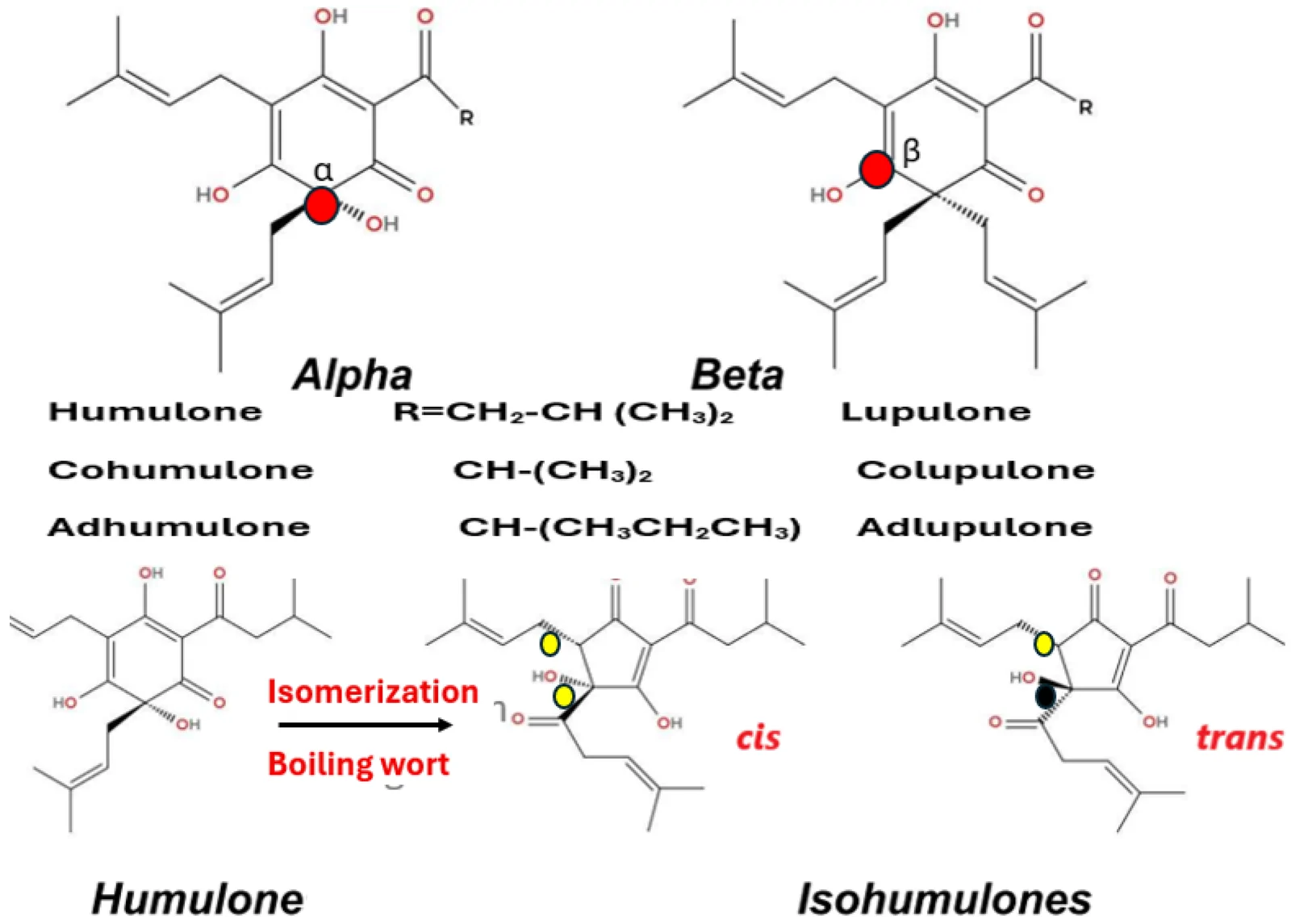
Molecular structures of alpha and beta acids (**top**). α and β carbons in red. Isomerization of humulone into cis/trans-isohumulone—yellow/black (**bottom**). Own elaboration based on PubChem.

Within the soft resin fraction, α- and β-bitter acids appear, respectively. The α-acids (humulones) constitute the most significant portion of soft resins. On average, they comprise between 9% and 10% of the α-acid weight, although this percentage can reach up to 19% in certain varieties [58]. Humulones are primarily composed of a mixture of six analogous molecules: humulone (35–70% of total α-bitter acids), cohumulone (20–55%), and adhumulone (10–15%), to which other minor fractions of posthumulone, prehumulone, and adprehumulone must be added [59,60]. Soft resins are soluble in hexane but poorly soluble in water. However, the thermal isomerization of α-acids (humulones) during boiling causes a change in molecular geometry, leading to the formation of isohumulones, which are more polar and soluble: specifically iso-α-acids and trans-iso-α-acids. Similarly, there are six β-bitter acids (lupulones): colupulone, lupulone, prelupulone, postlupulone, and adlupulone [60,61,62,63,64,65] (Figure 3).

The *β*-bitter acids (lupulones) are poorly soluble in water and generally account for less than 5% of the dry plant matter, depending on the variety [65]. Similarly to humulones, lupulones are primarily composed of six analogous molecules: colupulone, lupulone, prelupulone, postlupulone, adlupulone, and adprelupulone [65]. Hop hard resins are oxidized by-products of the hop acids present in the lupulin glands and contain complex plant compounds, mainly prenylated chalcones and flavanones. Xanthohumol is the primary molecule within hard resins as a prenylated flavonoid and remains the most extensively studied of this group [66]. From a brewing perspective, researchers are currently studying additional components that may be critical to the final characteristics of the product [67,68].

**Figure 4:**
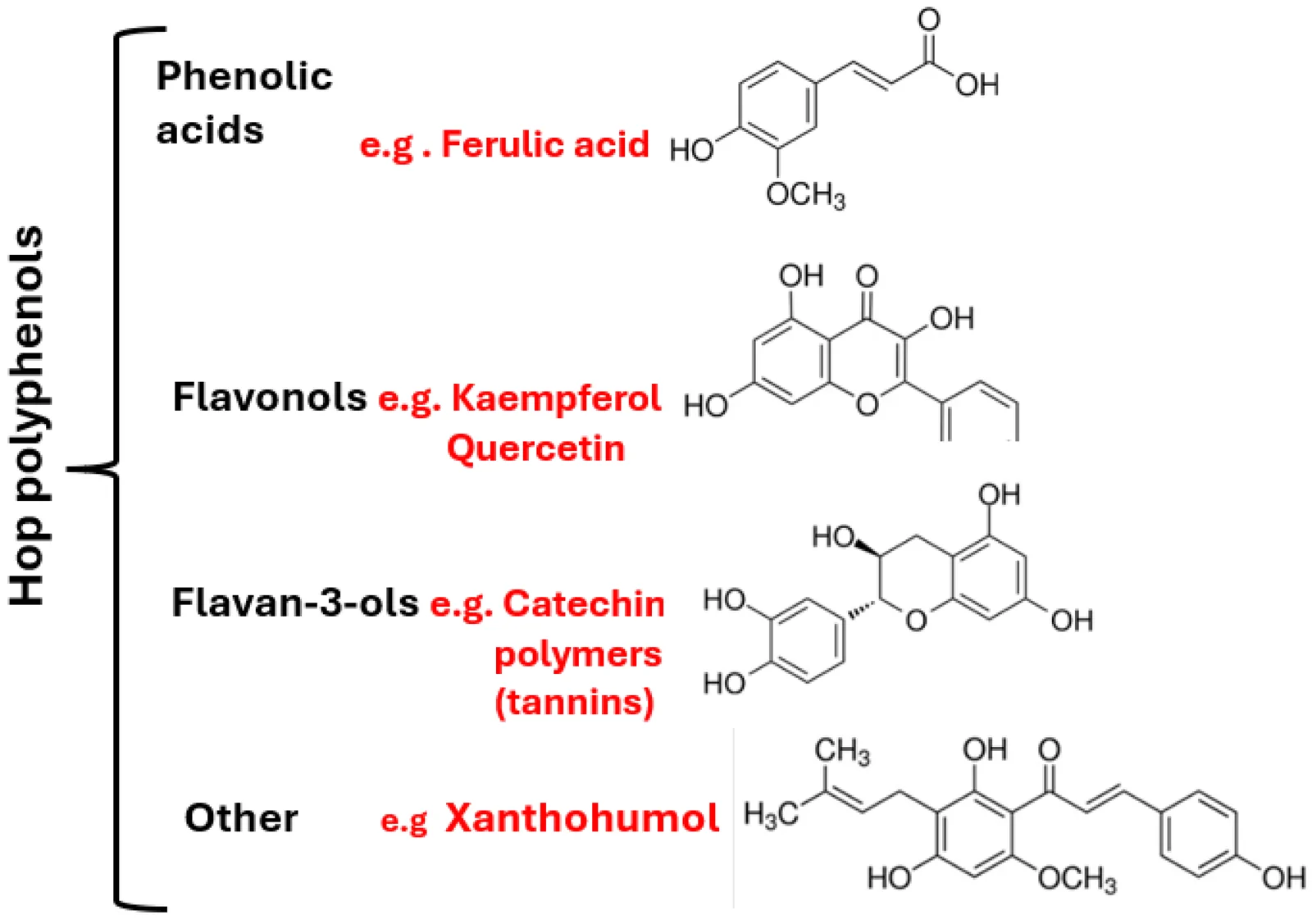
Different polyphenols present in the hops cones. Own elaboration based on PubChem.

Polyphenols (Figure 4) are secondary metabolites synthesized in plants and are generally useful against photodamage or pathogens. These phytochemicals have potential benefits to human health, mostly through antioxidants [69], which protect plants from reactive oxygen species and oxidative stress. This antioxidant activity can be measured using tools like ferric reducing antioxidant power, oxygen radical absorbance capacity and intracellular antioxidant [70]. Polyphenols do not dissolve during standard supercritical CO_2_ fluid extraction [71]. Instead, solvents such as ethanol–water mixtures or aqueous acetone are commonly employed to extract hop polyphenols, as the addition of water enhances the solubilization and extraction of these compounds [72,73].

The polyphenolic fraction of hops is so complex that researchers still continue to identify compounds [66]. Hop polyphenols are commonly classified into flavonols, flavan-3-ols (catechins), phenolic acids (benzoic and cinnamic acid derivatives), and other phenolic compounds such as prenylflavonoids and stilbenoids [74,75] (Figure 4). The majority of hop polyphenols are concentrated in the bracts and the rachis (strig) of the cone, whereas prenylflavonoids are secreted specifically by the lupulin glands. The principal prenylflavonoid is xanthohumol, alongside derivatives such as isoxanthohumol and 8-prenylnaringenin; these constituents, combined with resins and essential oils, account for between 4% and 14% of the dry matter of these glands and approximately 3% to 6% of the cone’s total dry weight [74]. Xanthohumol is considered exclusive to the hop plant [75,76]. These prenylated flavonoids serve as the primary active ingredients in various traditional Chinese medicines and functional foods, including the fruits of *Morus alba*, *Glycyrrhiza uralensis*, *Humulus lupulus*, *Artocarpus heterophyllus*, *Glycine max*, and *Ficus carica* [77].

**Figure 5:**
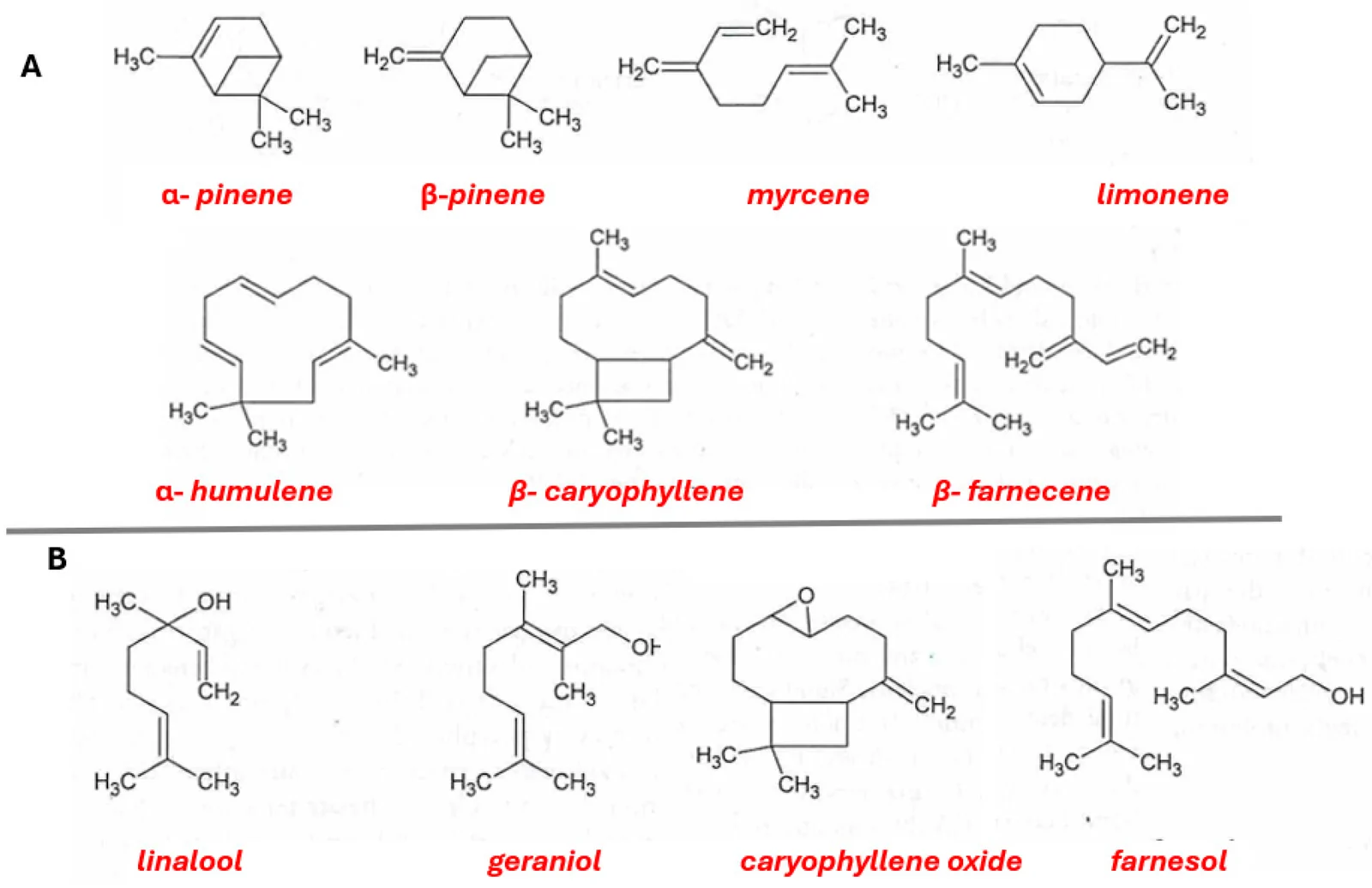
Some molecular structures present in hops essential oils: (**A**) hydrocarbures; (**B**) oxygenated compounds. Own elaboration based on PubChem.

Essential oils are complex, natural metabolic secretions that evaporate rapidly and are responsible for aromatic fragrances [78] (Figure 5). Some are known for their anti-inflammatory, antioxidant, antibacterial, and anticancer properties [79]. Common extraction methods include hydrodistillation, steam distillation, solid-liquid extraction, solvent extraction, cold pressing, supercritical fluid extraction, enzymatic action, and microwave-assisted extraction [80]. For the identification of molecules, gas chromatography (GC) coupled with mass spectrometry (MS) is typically used. For example, in the case of hops, 72 molecules were distinguished using Headspace Solid-Phase Microextraction and Gas Chromatography–Mass Spectrometry [81], while 208 molecules were identified using Gas Chromatography—Flame Ionization Detection and Mass Spectrometry [82]. Trials have also been conducted using gas chromatography-olfactometry (GC-O) to determine specific aromas [83].

In hops, the essential oils secreted by the lupulin glands, alongside bitter acids, represent between 0.5% and 3.0% of the dry hop mass. Approximately 300 different molecules have been identified, although the three primary ones are myrcene (68%), α-humulene (13.3%), and β-caryophyllene (3.7%) [84]. The chemical composition and essential oil content can be affected by the hop variety, cultivation conditions, maturity at the time of harvest, drying conditions, exposure to atmospheric oxygen, and storage conditions [81]. The chemical compounds present in hops essential oils are divided into three classes: hydrocarbons (50–60%), oxygenated compounds (30%), and sulfur-containing compounds (1%) (Figure 5). Hop hydrocarbons are primarily monoterpenes (α- and β-pinene, myrcene, limonene, etc.) and sesquiterpenes (α-humulene, β-farnesene, β-caryophyllene, etc.). Oxygenated compounds consist of a complex mixture of alcohols, aldehydes, ketones, epoxides, and esters (linalool, geraniol, caryophyllene oxide, farnesol, etc.). Examples of sulfur compounds include thiols, polysulfides, S-methyl thioesters, etc. [84,85,86,87].

### 2.3. Cultivation and Diseases

**Figure 6:**
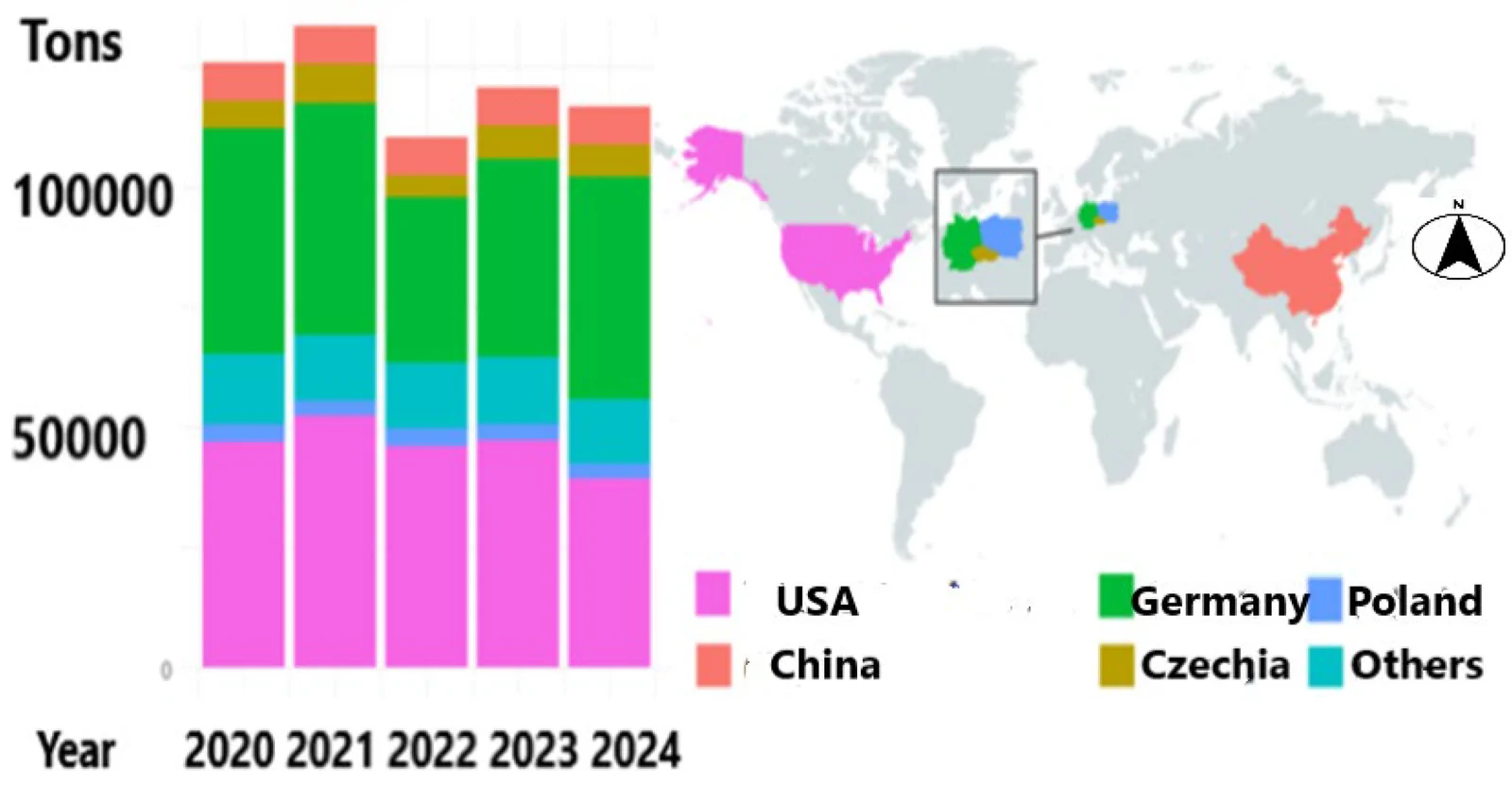
Metric tons of hops harvested in the top five producers. United States and Germany individually produce more hops annually than the rest of the world combined. Mape: [39].

Although *H. lupulus* is native to the Northern Hemisphere, it has been introduced and naturalized in multiple countries across both hemispheres, particularly within latitudes 35° and 55°. As an essential plant for the brewing industry, approximately 98% of global hop production is dedicated to beer. However, global production is highly asymmetrical: the market is dominated by the USA and Germany, which together account for 75% to 80% of the world’s total yield. In recent years, total global production has stabilized between 120,000 and 130,000 tons annually. Other significant producers include China, the Czech Republic, and Poland (Figure 6) [88]. Ethiopia is often excluded from standardized hop datasets because its reports frequently mislabel *Rhamnus prinoides* L’Hér. as “hops”; this is a native shrubby species used in traditional local brewing (tella and tej) instead of *H. lupulus* [89].

*H. lupulus* propagates through rhizomes which display usually in lanes (rows), utilizing trellis systems or support structures to sustain their climbing habit, reaching heights up to twelve meters [90]. Since the valuable compounds are exclusive to the female flowers (strobili), male plants are usually kept in isolation for seed production when necessary to facilitate proper cultivation in potentially less suitable zones [91]. In addition, cross-pollination would be risky, and could result in hybrid seeds with unknown genetics and probably unwanted, leading to the loss of the specific organoleptic profile required for specific beer styles. An individual’s sex cannot be identified at the seed stage, and since the plant’s commercial utility lies in female specimens, the accidental cultivation of male plants would represent a potential waste of resources. Therefore, there is a growing interest in developing and utilizing sex-linked DNA markers to identify the plant’s sex as early as the seedling stage [92].

Hops are harvested at the end of summer once the female flowers have bloomed and matured, and the lupulin glands reach their peak metabolite concentration. The collection should be conducted quickly because metabolite levels can decrease if the harvest is delayed [53]. Collection was traditionally performed by hand [13], but the process is now mechanized. The aerial section of the plant is cut by machines and taken to a processing facility to be dried until it reaches 9–11% humidity, as fresh cones on the plant contain around 75–80% moisture [90]. Drying is typically accelerated using kilns (ovens) or specialized drying areas at 45–60 °C; subsequently, air jets are used to separate leaves from cones. Cones are packed according to quality or are ground and compressed into pellets [53]. Pellets preserve flavor, aroma, and quality for longer periods [93].

Plant growth from rhizomes is remarkably fast, potentially reaching 5–6 m in just two months [94]. After winter dormancy, buds begin to swell and sprout [95]. The maximum development rate is reached by late June, with growth reaching up to 30 cm per day; it is around this time that the plants typically reach the top of the support structures [96]. *H. lupulus* exhibits strong apical dominance and begins to grow lateral branches once a substantial height is reached. Flowering occurs in mid-July for approximately ten days, and cones ripen from late August to early September, marking the start of the harvest season [30]. During the first year of growth, flowering is scarce or non-existent, and the plant is generally not cut to promote healthy root development; consequently, yield reaches its commercial peak during the fourth year. Plants are usually resilient and can typically be exploited for a period of 12 to 15 years, though individuals can live for up to 25 years. During cone ripening, the content of alpha acids increases, which is the most valuable component for brewing [30].

Certain environmental conditions are critical for hop cultivation, while others are less significant; this species thrives in moderately wet climates with temperate summers. The region must provide a substantial photoperiod and sufficient precipitation. Between harvest seasons and before sprouting, hops require more than 600 h of cold below 7 °C; although rhizomes are highly resistant to low temperatures, they cannot withstand conditions below −20 °C. Sprouting can occur below 0 °C, but the optimal temperature for sprouts is above 10 °C. Leaves require approximately 15–18 °C for optimal growth, and lateral branches develop best between 15 and 20 °C; however, they can tolerate low temperatures down to −2 °C. The ideal range for blooming is 20–24 °C, while cone ripening occurs optimally between 18 and 22 °C. The upper limit for heat tolerance is around 30 °C, but to endure such conditions, plants must be watered daily [30,90].

On the other hand, wind, dry soils, heat waves, and salinity are critical factors for *H. lupulus* during its early development after sprouting, as young shoots can be easily damaged [90]. In lower latitudes, rhizomes should not be planted late in the season, as higher temperatures may prevent the root system from establishing correctly to compensate for transpiration. Given that climate models predict more frequent droughts, *H. lupulus* shows high sensitivity to water deficits and can react quickly even to short periods of water scarcity, resulting in lower yields (up to 63% less production in European harvests) [97]. Soil acidity is also a restrictive factor; the optimal pH is between 6.0 and 6.5, and for values below 5.5, it is recommended to apply CaCO_3_ supplements to correct acidity and reduce aluminum saturation [30]. Hops require permeable soils for the correct development of their root systems, which can reach depths of 2 m [98]. Ideal soil types include loam, sandy loam, or clay loam. Typically, a distance of 3–4 m is left between rows to avoid excessive humidity, and a trellis system is constructed approximately 5.5–7 m high [30].

The salinity of both soil and irrigation water should remain below 4 dS/m both soil and water must be under 4 dS/m [99]. Altitude itself is not considered a direct limiting factor, and success has been reported at 3500 m above sea level [100]; however, high altitudes are usually colder and more moist, which are favorable conditions for *H. lupulus*.

**Figure 7:**
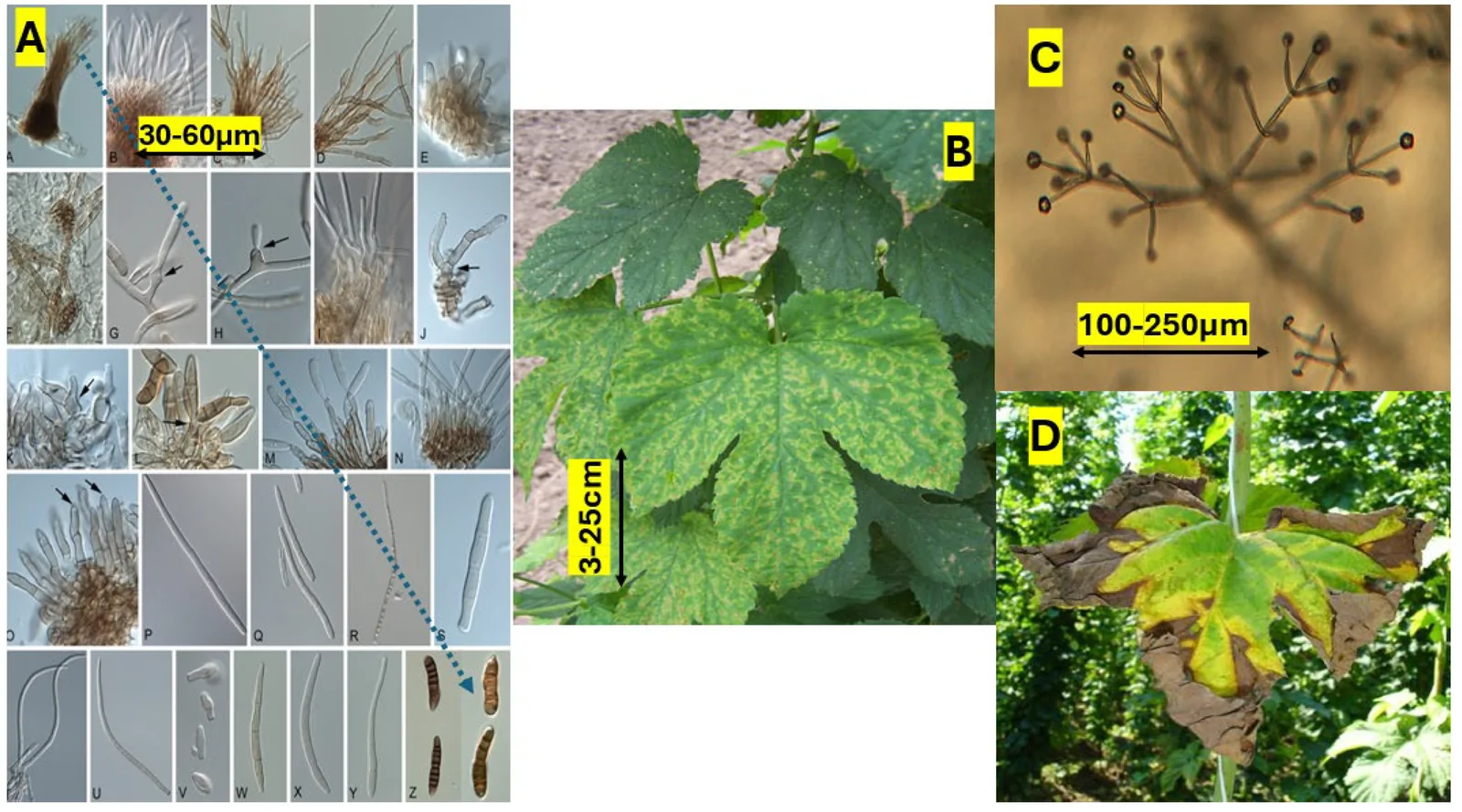
Hop pathogens. (**A**) Complete development of *Pseudocercospora* sp., including the stroma, conidiophores, hyphae, and conidiospores. (**B**) Variegated form of an infected leaf by virus. (**C**) Conidiophore of *Verticillium albo-atrum.* (**D**) Leaf with symptoms of *Verticillium* wilt. Compiled by the authors, with images for scientific use or reproduced with permission from the original authors: [123,124,125].

Hops can be attacked by a wide diversity of microbes living in the rhizosphere (bacteria, fungi, and nematodes) [101] and in plant debris or waterlogged areas (viruses, insects and arachnids) [102,103]. The effects of these pathogens vary: they can produce root tumors, inhibit proper development, and cause wilting or leaf chlorosis due to chlorophyll destruction, which translates into lower crop performance (Table 2, Figure 7). These are general aspects of hop phytopathology, for which it is fundamental to understand pathogen biology and disease ecology in depth [104].

From the list of all diseases (Table 2), wilt caused by *Verticillium albo-atrum* and *Verticillium dahliae* stands out as the most dangerous, as both produce survival structures in the soil [105,106]. *V. albo-atrum* produces dark mycelia, while *V. dahliae* produces microsclerotia. The cycle of *V. dahliae* begins when microsclerotia present in the soil (which are viable for several years) germinate in the presence of moisture; the hyphae penetrate the plant through roots or wounds and reach the xylem, spreading the fungus throughout the entire plant and reaching the leaf, where the characteristic conidiophore is produced. Symptoms of wilting appear, followed by the death of the plant, upon which new microsclerotia will develop, thus closing the biological cycle [107]. The severity of *Verticillium* wilt depends on the degree of individual infestation and the fungal strain [105]. Another noteworthy disease is caused by *Pseudoperonospora humuli*, a biotrophic oomycete (in this case, an obligate parasite of living plant tissues) that causes downy mildew, one of the most devastating diseases of cultivated hops. Downy mildew occurs in all production areas of this crop in the Northern Hemisphere and Argentina. The pathogen overwinters in the hop crowns and roots, causing considerable harvest losses [108,109].

**Table 2:** Some pathogens, effects and symptoms.

Species	Taxa	Effects and Symtopms	Refs.
*Pseudoperonospora humuli*	Oomycota	Causes hop mildew, an important disease in most hop-producing regions of the Northern Hemisphere. The pathogen overwinters in dormant crown buds and becomes systemic throughout the plant in spring, resulting in flower abortion.	[99]
*Podosphaera macularis*	Ascomycota	It causes powdery mildew of hop. Disease symptoms initially appear as small, white, powdery lesions, typically on the adaxial surface of the leaves, which subsequently develop into coalescing colonies on leaves, stems, and buds. A white powdery coating can also be observed on leaves and cones, reducing photosynthetic activity and potentially causing plant deformation.	[108,109,110]
*Verticillium dahliae*, *V. nonalfalfae*	Ascomycota	*Verticillium* wilt (VW) is one of the most serious soil-borne diseases. Colonization of host plants occurs through the root epidermis and, when successful, the fungus subsequently spreads into the vascular tissues, leading to systemic infection of the plant. Although disease symptoms vary among host species, they generally include wilting, chlorosis, necrosis, and vascular discoloration.	[111]
*Fusarium* spp.	Ascomycota	Fusariosis: attacks the vascular tissues and the plant crown, disrupting the flow of water and nutrients. Cankers and swelling develop at the base of the branches, which detach or break away from the crown with extreme ease when pulled. This results in progressive foliar chlorosis and necrosis.	[112]
*Botrytis cinerea*	Ascomycota	Noble rot attacks cones through wounds and duringhigh-humidity conditions, making it unusable. The *Botrytis* fungus occurs more frequently in years with frequent precipitation during the flowering period. Only the cones are affected. Individual bracts or cone tips turn a reddish-brown color.	[113]
*Alternaria alternata*	Ascomycota	Alternariosis: cause necrotic lesions at the tips of the bracts and bracteoles of developing cones, which may progressively extend throughout the entire cone. This results in production yield losses.	[114]
*Tetranychus urticae*	Arachnida	It is a generalist polyphagous pest that feeds on leaves. When feeding on hop cones, it damages the lupulin glands, resulting in reduced α-acid concentrations.	[115]
*Phorodon humuli*	Aphididae	Sap-sucking insects. Suck thes young parts of the plant andare. ector of viruses. They causethe leaves to become brittle. They can also attack the flowers.	[116]
*Psylliodes attenuata*	Coleoptera	A highly damaging beetle pest of hop crops. In spring, it feeds on basal leaves, causing defoliation and delaying plant development. Later, it attacks the cones, causing damage such as perforations and wilting.	[117]
*Xylena exsoleta*, *Anglais io*, *Polygonia c-album*	Lepidoptera	Insects whose larvae (caterpillars) are highly polyphagous and can cause complete defoliation of the plant.	[99]
*Heterodera humuli*	Nematoda	An obligate soil-dwelling parasitic nematode that affects the roots, potentially causing a significant reduction in cone yield	[118]
*Rhizobium radiobacter*	Bacteria	A soil-borne bacterium that induces tumor formation in the roots, thereby impairing the uptake of water and mineral nutrients, resulting in abnormal plant development and reduced yield.	[119]
Apple Mosaic Virus (ApMV)	Virus	It causes yellow or ring-shaped lesions on the leaves, which may progress to necrosis and potentially lead to plant death. In the cones, the concentrations of α- and β-acids may be drastically reduced.	[120]
Hops Mosaic Virus (HpMV)	Virus	It is a virus that is generally asymptomatic; however, some susceptible cultivars may develop severe foliar damage and impaired plant growth. Yield losses of up to 50% have been reported.	[120]
Arabis Mosaic Virus (ArMV)	Virus	Infected hop plants exhibit a range of characteristic symptoms, including reduced shoot production, shortened internodes, decreased leaf number, increased leaf fragility, and yellowish leaf mottling. In addition, infected plants show reduced growth rates and diminished climbing capacity.	[121]
Hops latent virus (HpLV)	Virus	It is generally asymptomatic; however, some cultivars may sporadically exhibit systemic chlorotic mottling.	[122]
Hop stunt viroid (HSVd)	Viroid	The disease induces a marked reduction in vegetative growth, characterized by shortened internodes and stems, chlorotic foliage, and the production of fewer and smaller cones. Consequently, cone yield and α-acid content may be substantially reduced.	[122]
Citrus bark cracking viroid (CBCVd)	Viroid	Similar to those caused by Hop stunt viroid (HSVd).	[122]

### 2.4. Utility and Usefulness

#### 2.4.1. Beer

Hops are primarily cultivated for the brewing industry [125], as they were an obligatory and irreplaceable ingredient for any beverage to be labeled as “beer” under the historical *Reinheitsgebot* purity law [12]. Although modern pasteurization and improved storage techniques have reduced the critical dependency on hops as a preservative, they remain an essential component. In contemporary brewing, hops are added for multiple reasons:

Bitterness (Flavor): The lupulin glands within the hop cones provide bitterness; when boiled with the wort, α-acids undergo thermal isomerization into bitter compounds that balance the sweetness of the malt [65].

Scent: In addition to resins, volatile essential oils produce diverse aromatic profiles, which are the identity of some beer types, and sometimes other aromatizers are used as well [65].

Foam formation and stability: Hop acids act as surfactants that help stabilize the bubbles, allowing the foam head to be more persistent and toadhere to the glass (a phenomenon known as lacing) [65].

Clarification: During boiling, hops help precipitate protein substances and certain nitrogenous compounds, which facilitates the subsequent clarification and filtration of the liquid [53].

In the European Union, there is no single community law that defines beer. In Spain, the legal definition is “food resulting from the fermentation, by means of selected yeasts, of a brewing wort prepared from natural raw materials” [126]. However, this five-page text explicitly prohibits the substitution of hops or their derivatives with other bitter principles and identifies hops as the botanical ingredient responsible for the bitterness, aroma, and natural protection of the beverage. Generally, beer brewing is primarily a biochemical and enzymatic process that also integrates plant science, microbiology, chemistry, process control, and flavor evaluation. The process centers on four main raw materials: barley malt and other cereals, hops, water, and yeast [127].

Iso-α-acids constitute the quantitatively most important hop-derived fraction in beer (Table 3). Along with CO_2_ and ethanol, they are considered the primary components of beer flavor. They provide the characteristic bitter taste, equivalent to that of quinine (a bitterness standard), depending on their concentration, which varies between 10 and 100 ppm [128,129]. This bitter taste is primarily provided by iso-α-humulones (mainly iso-α-cohumulones; Figure 3), resulting from the thermal isomerization of humulones, which balance the sweetness of the malt [130].

Although modern pasteurization and improved storage techniques have reduced the critical dependence on hops as a bactericide derived from its bitter acids (and thus as a product preservative), iso-α-acids exhibit significantly better bacteriostatic properties than iso-β-acids, which are present in beer in very small amounts [131]. Beer, in fact, must be preserved in opaque packaging to prevent UV light and riboflavin (vitamin B12) present in the beverage from reacting with iso-α-acids (light-struck effect), which generates a chemical compound (MBT) with a very unpleasant odor [132].

Iso-β-acids degrade during the boiling process of beer production and do not contribute bitterness during boiling; instead, they oxidize during prolonged storage or aging, adding unpleasant organoleptic characteristics. Consequently, hop varieties rich in β-acids are less appreciated by brewers [4,63]. Some hops are used solely to provide flavor and aroma to the beer, while others provide bitterness and stability. Lately, many hops have been classified as “dual-purpose or mixed,” meaning they can fulfill both functions. The type of hop is very important when choosing when to use it in a recipe, as it determines the beer’s profile and flavor. According to the α/β acid ratio of the hop in question, these can be classified into bitter (2:1 or 3:1; 10–20% α-acids), aromatic (1:1; 2–10% α-acids), or mixed (high proportion of α-acids, 10–15%, and essential oils) [133].

Beer contains significant levels of polyphenols (between 20 and 30 ppm), of which approximately 70–80% come from barley malt and 20–30% from hops [134]. After adding hops, it has been verified that quercetin concentrations increase while catechin levels remain nearly constant [135]. The role of these molecules in the long-term preservation of beer is debated, as they can cause turbidity through polyphenol condensation and produce off-flavors; thus, they are typically minimized during the manufacturing process [136,137]. Even so, polyphenols beneficial to health can be found in beer originating from hops, such as: ferulic acid (≤6.5 ppm), kaempferol (16.4 ppm), quercetin (≤10 ppm), catechins (≤5.4 ppm), xanthohumol (0.002–1.2 ppm), etc. [138].

Volatile essential oils are responsible for beer aroma; numerous molecules have been identified in beer, which may exert a synergistic effect with one another to provide the final flavor profile of the product [81]. There are several characteristic molecules common across different hop varieties, attributing various aromatic traits to them, such as: β-myrcene (herbaceous, resinous, balsamic, fresh hops), α-myrcene (resin, pine, herbal), α-humulene (spicy, woody), β-caryophyllene (woody, spicy), β-farnesene (woody, sweet citrus), α-pinene and β-pinene (pine), linalool (floral and fruity notes, citrus, bergamot), and caryophyllene oxide (sweet, fruity, woody, and herbal), among others [135,139].

Some Ancient beliefs regarding the medicinal effects of hops whose veracity has been demonstrated, such as its antiseptic properties or its use as an alternative to opium treatments due to its sedative effects, lacking the adverse side effects (headache, stomach pain, constipation) associated with the latter [15]. As mentioned in the introduction, pillows filled with hops are marketed today. Hops act as a sedative for the central nervous system. Specifically, hop components such as humulone and xanthohumol bind to and modulate γ-aminobutyric acid receptors (an inhibitor of neuronal activity), which helps create a calming, sedative, and sleep-promoting effect [139]. In the past, treatments with hops were designed to counter excesses of black bile (within the humorism framework), and were later used as diuretics, promotinf kidney and stomach health [15]. However the diuretic property can be controversial; hops are diuretic in isolation, but if they are consumed through beer, theycan produce kidney stones (because of the high quantity of purines from the yeast), which induces the production of uric acid [140].

#### 2.4.2. Medicinal Properties

Hops have been used since ancient times for their medicinal properties (see Section 1). Currently, standardized hop extract is legally marketed in Spain for oral administration in the form of capsules or infusions at concentrations of 125 mg per dose. The General Council of Official Colleges of Pharmacists (CGCOF) of Spain currently lists 77 products containing *H. lupulus* among their ingredients, including serums, lotions, balms, and tablets, sometimes in combination with *Valeriana officinalis* L. These products are indicated to improve sleep quality, alleviate anxiety, and stimulate appetite (orexigenic effect) [141].

Prior to their validation and commercialization, numerous studies investigated the bioactive compounds of hops and their potential applications. Hop extract exhibits antibacterial and antifungal activities, primarily attributed to β-acids and prenylated flavonoids, particularly xanthohumol, with particularly potent activity against Gram-positive bacteria. Antifungal activity has also been demonstrated against several pathogenic fungi, including *Trichophyton* sp., *Mucor rouxianus*, and *Fusarium oxysporum* [142]. Furthermore, xanthohumol has attracted considerable interest because of its potent antioxidant and anti-inflammatory properties [143], and has been shown to exert toxic effects against *Plasmodium falciparum*, the protozoan parasite responsible for the most lethal form of malaria in humans [144] (Table 3).

Xanthohumol also appears to hold considerable promise for future therapeutic strategies. This compound exhibits notable anticancer potential, promoting apoptosis in several cancer types, including breast, colon, and prostate cancer, while inhibiting the growth and proliferation of malignant cells [142]. Bitter acids have demonstrated promising antiproliferative activity against multiple cell lines in vitro, while exhibiting relatively low toxicity toward healthy cells [34,145]. Finally, dietary supplementation with hop-derived compounds may be effective as an adjuvant approach in the management of metabolic syndrome, owing to their hypoglycemic, hypolipidemic, anti-obesity, and cardiovascular effects, which have been associated with xanthohumol and bitter acids [146]. For a more comprehensive review of this topic, we recommend four particularly informative reviews by Karabin et al., Carbone et al., Knez Hrnčič et al., and González-Salitre et al. [31,138,145,147,148], which extensively examine the relationships between bioactive hop compounds and various diseases, highlighting the potential of this plant as a source of compounds of interest for human health.

Phytoestrogens is a general term used to describe structurally diverse compounds of plant origin that mimic or modulate the actions of estrogenic hormones through their ability to interact with estrogen receptors. Hops contain some of the most potent phytoestrogens identified in nature to date [149]. Xanthohumol, which has been mentioned several times throughout this review, is one such compound. Following metabolism by the human body and intestinal microbiota, xanthohumol can be converted into other compounds, including isoxanthohumol and 8-prenylnaringenin (8-PN). The intestinal absorption and systemic bioavailability of these compounds are limited, partly because of their hydrophobic nature. Therefore, the development of novel pharmaceutical formulations and delivery systems may substantially improve their stability and increase their plasma concentrations [105,150].

The primary phytoestrogen found in hops is the prenylated flavonoid 8-prenylnaringenin (8-PN) [151]. Available evidence suggests that dietary intake of these compounds may provide therapeutic benefits, particularly in alleviating postmenopausal symptoms and reducing the risk or progression of atherosclerosis. Accordingly, hop-derived phytoestrogens have been proposed as natural alternatives to hormone replacement therapy (HRT) to compensate for declining endogenous estrogen levels. Nevertheless, the estrogenic potency of these compounds remains lower than that of human 17β-estradiol [152]. However, some authors have raised concerns that the estrogenic activity of 8-PN may lead to undesirable effects following prolonged supplementation [149,150].

Hop essential oils exhibit analgesic, anticancer, sedative, and anti-inflammatory properties [66]. These oils may be used in formulations intended for the management of various skin conditions, including acne, psoriasis, and dermatitis [153]. Furthermore, certain Brazilian varieties of *H. lupulus* have demonstrated photoprotective potential, showing a significant capacity to enhance protection against solar radiation, with reported sun protection factor (SPF) values of up to 39 [154]. Other compounds of interest include myrcene, linalool, and geraniol (Figure 5), which are commonly incorporated into cosmetic formulations because of their specific aromatic and potentially therapeutic properties [154,155].

#### 2.4.3. Cosmetics

*H. lupulus* is used as an ingredient in a variety of cosmetic products, some of which may also be classified as cosmeceuticals. *H. lupulus* extract [156] exhibits a broad range of properties that may positively influence skin condition and function. Its documented sedative and anti-inflammatory activities, together with its ability to reduce skin reactivity, make this extract of particular interest for cosmetic formulations intended for sensitive and highly reactive skin [157].

The antioxidant potential of hops is particularly relevant to dermatology, as hop-derived compounds have been shown to protect cells against oxidative stress induced by ultraviolet (UV) radiation and environmental pollutants, thereby potentially helping to prevent premature skin aging and collagen degradation [158] (Table 3). This antioxidant activity has been primarily attributed to flavonoids such as xanthohumol, isoxanthohumol, and 8-prenylnaringenin, as well as other phenolic compounds and glycosides, including quercetin and kaempferol [138]. In particular, xanthohumol inhibits elastase and tyrosinase, enzymes directly involved in skin degradation and the formation of hyperpigmented lesions [159].

**Table 3:** Some biological and industrial activities of hops.

Molecules	Activity	Refs.
**α-acids**	Hey provide bitterness and contribute to the formation and stability of beer foam. They exhibit antimicrobial effects, particularly bacteriostatic activity, by inhibiting the growth of Gram-positive bacteria. Dietary supplementation with hop-derived compounds may be effective as an adjunctive approach in the management of metabolic syndrome, owing to their hypoglycemic, hypolipidemic, anti-obesity, and cardiovascular effects, which have been associated with xanthohumol and bitter acids.	[12,81,137,139,153,154,155,156,157,158]
**Polyphenols**	Beneficial to health due to their antioxidant properties. Ferulic acid, kaempferol, quercetin, catechins, and xanthohumol, among others. Prenylated flavonoids (xanthohumol). Activity against Gram-positive bacteria. Antifungal activity against *Trichophyton* sp., *Mucor rouxianus* and *Fusarium oxysporum*. Activity against *Plasmodium falciparum* (the causative agent of malaria). Produces a calming, sedative, and sleep-promoting effect through binding to gamma-aminobutyric acid receptors. Oncological potential, as it promotes apoptosis in various types of cancer (including breast, colon, and prostate cancer). Phytoestrogenic capacity with potential benefits for reducing postmenopausal symptoms and atherosclerosis.	[138,142,143,144,145,151,152]
**Essential oils**	Essential oils contribute to the aroma profile and final flavor of beer and include α-myrcene, β-myrcene, β-caryophyllene, β-farnesene, α-pinene, β-pinene, linalool, and caryophyllene oxide. Myrcene, linalool, and geraniol are commonly used in cosmetic formulations because of their aromatic and cosmeceutical properties. These compounds are also incorporated into formulations intended for the management of acne, psoriasis and dermatitis. In addition, they may exhibit photoprotective activity against solar radiation.	[12,81,135,139,153,158]

#### 2.4.4. Toxicity

*H. lupulus* is a plant with low toxicity, and it is not in general considered a dangerous plant. When consumed through beer, the quantity taken is low, and the concern should be directed towards the alcohol ingested or the purines from the yeast instead of hops, which have a large list of risks. However, high doses of hops supplements may have certain negative effects on certain human groups. Phytoestrogens can pose risk and can stimulate estrogenic-positive tumors, therefore, hops consumption is not recommended for people at risk of breast or endometrial cancer, nor for survivors of these cancers [160,161]. It is also advised that it should not be taken during pregnancy and lactation [55]. Some problems of fertility have been observed in pigs, as 8-PN can affect the formation of the spindle apparatus inovocyte maturation, which could lead to infertility problems [162]. There is also historical evidence that high exposure to the plant itself can lead to menstrual cycle irregularities, as menstrual bleeding has been reported, two days after the harvest season started [163], in addition to drowsiness during the collecting days [24]. Despite xanthohumol being highly valued as an antioxidant [141], at higher concentrations, can cause the opposite ofthe desired effect, namely and oxidative damage in the DNA [34]. Despite the low toxicity of *H. luplulus*, high intake concentrations have been found to be lethal in animals: 62.5 μg/mL in fruit flies [164], and LD50 of 175 mg/kg in lab mice [165]. However, there is no known lethal dose in humans; in fact, hops are labeled as a GRAS product by the U.S. Food and Drug Administration, which means that they are safe to ingest [166].

## 3. Conclusions

This cross-sectional review of various aspects of *H. lupulus* aims to provide a comprehensive understanding of this species from the varying perpestives of several disciplines, catering to both individuals with a general interest in the plant and researchers specializing in the study of its exclusive properties. Despite being a widely recognized species, there is still considerable debate regarding its botanical position and taxonomy, particularly concerning nomenclature and its extensive number of synonyms and varieties. Hops are irreplaceable in the brewing of beer (*sensu lato*),a role that has driven essential research into their chemistry. This is, a field that continues to be explored through advanced analytical tools and the recent integration of genomic and proteomic resources.

The use of standardized hop extracts represents a beneficial option for nutraceutical, therapeutic, and pharmaceutical applications aimed at preventing and, where applicable, treating chronic pathologies. This potential is supported by numerous studies linking various bioactive compounds (such as volatile oils, α/β acids, and polyphenols, specifically prenylflavonoids) to pleiotropic health benefits, given their antioxidant, antimicrobial, phytoestrogenic, sedative, and antiproliferative properties. Nevertheless, this potential must still be confirmed in human subjects rather than relying solely on in vitro assays or animal models. Simultaneously, the pharmaceutical industry is expected to develop targeted, non-toxic formulations that significantly enhance the bioavailability of the plant’s most significant components. Ultimately, hops remain an ancient species from both botanical and ethnomedicinal perspectives, maintaining great relevance for ongoing scientific analysis.

## Figures and Tables

**Table 1:** Average chemical composition of dry hop cones.

Compound	Value (%) [54] vs. Other papers [31,54,55,56]	Refs.	Compound	Value (%) [53] vs. Other papers [31,54,55,56]	Refs.
Resins	15–30 (10–15)	[54]	Amino acids	0.1 (0.1)	[54]
Essential oils	0.5–3 (0.45–2.7)	[55]	Waxes and steroids	traces (3)	
Proteins	15 (15)	[54]	Ash	8 (8)	[54]
Monosaccharides	2 (2.0)	[54]	Humidity	10 (10)	[54]
Polyphenols	4 (3–14)	[31]	Cellulose, etc.	43 (40)	[31,56]
Pectins	2 (2)	[54]			

## Data Availability

No new data were created or analyzed in this study. Data sharing is not applicable to this article.
